# Head CT Scans in the Emergency Department during the COVID-19 Pandemic: Use or Overuse?

**DOI:** 10.3390/life14020264

**Published:** 2024-02-17

**Authors:** Marcello Covino, Andrea Piccioni, Giuseppe Merra, Carolina Giordano, Rosellina Russo, Amato Infante, Luca Ausili Cefaro, Luigi Natale, Francesco Franceschi, Simona Gaudino

**Affiliations:** 1 Emergency Department, Fondazione Policlinico Universitario A. Gemelli, IRCCS, 00168 Rome, Italy; marcello.covino@policlinicogemelli.it (M.C.); andrea.piccioni@policlinicogemelli.it (A.P.); giuseppe.merra@policlinicogemelli.it (G.M.); francesco.franceschi@policlinicogemelli.it (F.F.); 2 Istituto di Radiologia, Università Cattolica del Sacro Cuore, 00168 Rome, Italy; luigi.natale@unicatt.it (L.N.); simona.gaudino@policlinicogemelli.it (S.G.); 3 Department of Diagnostic Imaging, Oncological Radiotherapy, and Hematology, Fondazione Policlinico Universitario A. Gemelli, IRCCS, 00168 Rome, Italy; carolina.giordano@guest.policlinicogemelli.it (C.G.); amato.infante@policlinicogemelli.it (A.I.); luca.ausilicefaro@policlinicogemelli.it (L.A.C.)

**Keywords:** COVID-19, head contrast CT, vaccine, cerebrovascular disease

## Abstract

Background: The COVID-19 pandemic seemed to mainly involve the respiratory system, but it was realized that it could affect any organ, including the CNS. The pandemic has followed a wave-like trend, with its peaks being due to the COVID-19 different variants and the introduction of the vaccine, which led to an apparent reduction in hospitalizations but also brought about perplexities related to its adverse effects. The aim of this study was to analyze the changes in the use of head CT/contrast CT and their impacts on the onset of cerebrovascular disease in our emergency department during the COVID-19 period and the vaccine rollout. Methods: Patients ≥ 18 years old admitted to our emergency department from January 2018 to September 2021 were enrolled. The patients were divided into three groups. The COVID-19 period included patients who visited our emergency department from 1 March 2020 to 31 January 2021; the vaccine period was considered to range from 1 February 2021 to 30 September 2021. The patients who visited the emergency department from 1 January 2018 to 31 January 2020 were considered the controls. Results: We found an increase in head CT/contrast CT requests during the COVID-19 period and increase in head contrast CT during the vaccine period, without an increase in the incidence of cerebrovascular disease. Conclusions: The uncertainty regarding the possible thrombotic events associated with COVID-19 and its vaccine increased the relative use of head CT/contrast CT by about 20% compared to the control period

## 1. Introduction

COVID-19 is a novel infection with severe clinical manifestations, including death, that has reached at least 124 countries and territories. The World Health Organization (WHO) officially declared the COVID-19 outbreak a pandemic on 11 March 2020 [1,2].

This infection is primarily known to cause severe breathing difficulties; however, in a few months after the start of the pandemic, it became clear that infection from COVID-19 could affect multiple organs, including central nervous system impairment [3,4].

The pandemic has followed an undulatory flow, with its peaks being due to the varying levels of aggressiveness of the different COVID-19 variants [5,6,7] and periods of reduced manifestation, which vaccination has fundamentally contributed to in Italy, with an evident reduction in hospitalizations being recorded after the vaccine rollout [7,8,9].

From the point of view of the clinical management of patients, the pre-vaccine period was undoubtedly the most complex for doctors and healthcare professionals; however, even during the vaccine period, in the initial phase, there were significant concerns about its adverse effects [10], even if it was soon shown that severe adverse events were unusual and that the benefits of vaccination against COVID-19 far outweighed the potential risks [11].

Although the ultimate course and impact of COVID-19 are uncertain, the disease has overwhelmed healthcare infrastructure worldwide [12]. In Italy, since February 2020, COVID-19 has placed extraordinary and sustained demands on health systems, with the consequent need, especially in 2020, to ration medical equipment and interventions [13].

During these years, our emergency department (ED) has undergone pivotal changes, as well as a significant decrease in the efficiency of certain services because of measures such as lockdowns and stay-at-home orders.

Healthcare workers had to manage an infection with an unclear etiology and pathology, along with possible multi-organ involvement and a high mortality rate, in conditions characterized by a fear of contracting the disease themselves and passing it on to their loved ones [14,15].

Because of this, although the movement restrictions generated by COVID-19 have reduced the overall radiological workload, this has corresponded to an increase in the number of studies being performed on the management of the COVID-19 infection, as well as the urgency with which it should be managed [16].

Herein, we report our experience in a second-level emergency department in Italy, Rome (a metropolitan city), to address whether and how the pandemic period and vaccine period modified the demand for resources—in particular, CT head scans—from the emergency department for acute cerebrovascular disease.

## 2. Materials and Methods

This study was a single-center, retrospective, cross-sectional study conducted in the ED of an urban teaching hospital, a referral center for COVID-19 in central Italy, and a referral center for acute stroke treatment.

The study enrolled all patients ≥ 18 years old admitted to our ED from January 2018 to September 2021. We excluded all the patients who accessed the ED for trauma- and pregnant-related reasons from our analysis.

The patients were divided into three groups for the analysis based on the date of their ED visit, and the COVID-19 period included patients who visited the ED from 1 March 2020 (the date of the first COVID-19 cases in our ED) to 31 January 2021. The vaccine period included patients who accessed the ED from 1 February 2021 to 30 September 2021. Patients who accessed the ED from 1 January 2018 to 31 January 2020 were considered as controls.

The electronic clinical records of all patients were reviewed to retrieve the following clinical and demographic data:

- Age and gender.
- ED presentation data, including the date of ED access, assignment of high-priority triage code, and transportation to the ED by the emergency medical service
- The presentation of symptoms and clinical findings, including neurological deficit(s) (any), alteration in consciousness, epilepsy, confusion, headache, sensitivity disorders, vertigo or dizziness, malaise or fatigue, vomiting, and syncope or presyncope.
- Clinical history and comorbidities, particularly regarding cardiovascular risk factors.

These factors included a history of cerebrovascular disease, peripheral vascular disease, hypertension, lipid disorders, coronary artery disease, diabetes, chronic kidney disease, active anticoagulant therapy (either vitamin K antagonist or direct anticoagulant), and active antiplatelet therapy.

- The radiological workup included requesting a head CT scan without or with contrast media in the ED.
- Based exclusively on the ED discharge diagnosis, we retrieved the cerebrovascular disease diagnoses, including those for ischemic stroke, cerebral hemorrhage, and cerebral vein thrombosis.

The CT and CTA exams were performed by using a 64-slice CT scanner (Revolution EVO, GE Medical System, Chicago, IL, USA) and a 128-slice CT scanner (Otima CT 660, GE Medical Systems, Chicago, IL, USA). The CT images, with a slice thickness of 2.5 mm, SFOV head, and a matrix of 512 ×  512, were obtained in axial mode. Head CT exams in our emergency department were most commonly performed as a non-contrast study; the addition of a contrast-enhanced phase is performed mainly for cerebrovascular indications. CTA was performed following the injection of 60–140 mL of a non-ionic contrast agent (Iohexol, GE Healthcare, Chicago, IL, USA) at a rate of 3–4 mL per second. The median parameters were 1.25 mm section thickness, 0.5 mm intersection gap, and 512 ×  512 matrix. The axial, sagittal, and coronal CTA images were reconstructed with a slice thickness of 2 mm and an intersection gap of 1 mm.

### 2.1. Study Endpoints

The primary study endpoint was the evaluation of the overall rate of prescription of head CT scans and contrast head CT scans in the ED for acute cerebrovascular diseases in three periods: the COVID-19 period, the vaccine period, and the pre-COVID-19 period. As a secondary endpoint of the study, we evaluated the rate of cerebral vein thrombosis diagnosis in the ED.

### 2.2. Statistical Analyses 

Continuous variables are reported as median [interquartile range] and were compared by univariate analysis using the Mann–Whitney U test, or the Kruskal–Wallis test in cases involving three or more groups. Categorical variables are reported as absolute numbers (percentage) and were compared using a Chi-square test (with Fisher’s test if appropriate).

To correct the crude rate of head CT requests for the clinical factors potentially influencing the ED physician’s decision to prescribe (or not prescribe) neuroimaging, we entered all the factors presenting a univariate association with the performing of neuroimaging in the ED into a logistic regression model. The logistic regression results helped us to obtain adjusted odds relating to the neuroimaging requests based on the distribution of clinical and other contingent factors in the evaluated patients. 

To obtain adjusted odds of neuroimaging requests for the COVID-19 period and the vaccine period, we forced this variable in the logistic model, using the control period (2018–2019) as a reference. Similarly, to ascertain the changes in the adjusted odds of neuroimaging requests during the vaccine period, we entered a variable into the logistic model indicating the month of ED access in 2020. Even in this latter analysis, the control period was used as a reference for odds calculation.

The odds ratio was presented as OR [95% confidence interval]. A two-sided *p* ≤ 0.05 was considered significant in all the analyses. Data were analyzed using SPSS v25^®^ (IBM, Armonk, NY, USA). 

### 2.3. Ethics Statement

This study was conducted according to the Declaration of Helsinki and its later amendments and was approved by the local institutional review board, the Ethics Committee of Fondazione Policlinico Universitario A. Gemelli IRCCS (4/2020 no 3139).

## 3. Results

### 3.1. Patients

In the study period, 239,984 patients were evaluated in the ED. After the exclusion of patients with trauma-related issues, subjects < 18 years, and pregnant women, 167,549 patients were included in the analysis, with the average age being 60 (44–75) and the total number of males being 83,657 (49.9%). Overall, 99,962 patients were evaluated in the control period, whereas 37,303 and 30,284 were assessed in the COVID-19 and vaccine periods, respectively (Table 1 and Table S1). The median age of the treated patients was around 60 years in all three periods. However, the patients treated during the COVID-19 period and the vaccine period were slightly but significantly older (Table 1).

Overall, 24,566 head CT scans (14.7% of evaluated patients) and 2915 contrast head CT scans (1.7% of estimated patients) were prescribed in the ED. The rate of both CT prescriptions was significantly higher in the COVID-19 and vaccine periods compared to the control years. The crude ratio of stroke and cerebral hemorrhage diagnosis was lower in both pandemic periods compared to the control years. Given the increased utilization of emergency neuroimaging and the relative reduction in acute cerebral non-traumatic diseases, an acute pathological finding upon head CT was found in only 17.3% of patients in the vaccine period and 13.8% of patients in the COVID-19 period, compared to 25.3% in the control period (*p* < 0.001).

Conversely, the diagnosis of cerebral vein thrombosis was more common in the vaccine period, being found in 4.3/10^4^ patients compared to 1.8/10^4^ and 1.6/10^4^ in the COVID-19 and control periods, respectively (*p* < 0.001).

### 3.2. Factors Associated with Head CT Prescription in the ED

As expected, many different clinical and contingent factors were associated with a different prescription rate of a head CT scan in the ED. Older patients, patients accessing the ED through the emergency medical services, and patients with high-priority triage had a higher rate of CT scans (Table 2 and Table S2). Interestingly, independent from other clinical variables, these factors were associated with higher odds of head CT. Though not entirely explainable by other clinical findings, the male sex was associated with slightly but significantly higher odds of receiving neuroimaging in the ED. Most of the evaluated symptoms and clinical findings, as well as cardiovascular risk factors, were associated with different odds of receiving a head CT scan in the ED. As largely expected, acute neurological deficits and disturbances like confusion, headache, and alteration in consciousness were independently associated with higher odds of CT examination (Table 2 and Table S2). Among the less specific clinical findings, vertigo, dizziness, and syncope were associated with increased odds of CT examination.

Most cardiovascular risk factors were also associated with increased odds of head CT scans in the ED, except for lipid disorders, history of coronary disease, and diabetes (Table 2 and Table S2).

After adjusting for all the evaluated factors, the odds of receiving a head CT scan in the ED were about 20% higher in the COVID-19 period (OR 1.182). In contrast, it was similar to the control years in the vaccine period (OR 0.975) (Table 2 and Table S2).

### 3.3. Factors Associated with Contrast Head CT Scan Prescription in the ED

Overall, 2915 patients (1.7%) received a contrast head CT scan in the ED. Similarly to non-contrast examination, several factors were independently associated with contrast agent utilization for neuroimaging. In particular, older patients and patients visiting the ED due to a suspected time-dependent pathology (as evidenced by high-priority triage or transportation by the emergency medical services) had higher odds of a contrast CT evaluation. Similarly, neurological findings and signs associated with a higher risk of acute stroke (neurological deficit) were associated with the highest odds of a contrast CT evaluation (Table 3 and Table S3). Among the less specific signs and symptoms, headache presence was most commonly associated with a contrast examination. Notably, epilepsy and syncope were associated with reduced odds of a contrast examination. As expected, a clinical history of reduced kidney function and peripheral artery diseases were associated with lower odds of contrast agent utilization (Table 2 and Table S2). Though not completely explainable by other clinical findings, the adjusted odds for contrast head CT examination were about 10% higher in the COVID-19 period (OR 1.107) and about 30% higher in the vaccine period (OR 1.339) (Table 3). Upon conducting a sub-analysis to calculate the adjusted odds of contrast head CT over the months of 2021, we observed a trend for higher odds of utilization from February to June. In contrast, the adjusted odds decreased in the later months. However, during the whole of 2021 and the COVID-19 period, the adjusted odds for contrast CT scan prescription were more than 20% higher compared to the control years.

## 4. Discussion

The results of our study show a greater use of head CT and contrast CT during the COVID-19 and head contrast CT during the vaccine period than the control (pre-COVID-19) period. In the face of an additional risk factor, the final diagnosis of cerebrovascular diseases did not increase. 

Factors such as age, access to the ED via the emergency medical service, and triage scale had a similar impact on the probability of undergoing a head CT scan. Unsurprisingly, acute neurological deficits and disorders were independently associated with higher possibilities of CT examination across all periods. It is more surprising that, in all three considered periods, even less specific symptoms such as vertigo, dizziness, and syncope led to higher odds of head CT, given the fact that most guidelines recommend against this practice. 

Clinical factors associated with a higher prescription rate of a head CT scan in the ED were similar in the three periods, except for one factor: COVID-19 infection. The incidence of new-onset cerebrovascular diseases during COVID-19 infection ranged from 0.5% to 5.9%. An increased risk of cerebrovascular diseases with COVID-19 infection can have many adverse outcomes, resulting in increased morbidity and mortality compared with non-COVID-19-related cerebrovascular diseases. Thus, COVID-19 infection represented a risk factor that may have increased the odds of receiving a head CT scan during the COVID-19 period. However, this cannot be the only explanation, as the odds returned to figures similar to the control years in the vaccine period (OR 0.975). We could hypothesize that the need for neuroimaging support has been less felt over time in managing patients with brain pathologies.

As for the reduced odds of a head CT with contrast in cases of epilepsy and syncope, these data could be explained by our neuroradiology procedures. In the emergency department setting, CT with contrast media is mainly aimed at detecting vascular occlusions or malformations requiring time-dependent treatment. A contrast medium is not administered in the ED in cases of intracranial mass or basal ganglia hemorrhage.

Despite our prevalent use of contrast CT for acute cerebrovascular diseases in ED, it remains incompletely explainable why, despite a reduction in the diagnosis of cerebrovascular diseases, the adjusted odds for contrast CT were about 10% higher in the COVID-19 period and about 30% higher in the vaccine period. One hypothesis suggests a near collective hysteria fueled by national media regarding COVID-19 vaccination side effects. The potential thrombotic complications of the vaccine, ultimately proven to be rare and less frequent than those resulting from COVID infection [11], triggered considerable concern and even panic within the population and medical community. It has been reported that an awareness of treatment side effects can lead to the manifestation of similar symptoms [17]. In our view, this heightened attention from both the general population and physicians towards mild symptoms such as headaches was often interpreted as an early warning sign of a vaccine complication “until proven otherwise.” Consequently, headaches in vaccinated individuals were more frequently associated with contrast-enhanced CT examinations. 

During the vaccination period, two thrombosis diagnoses were denied in subsequent checks; they were both arachnoid granulations. This condition never occurred in the COVID-19 or control periods.

The variations in CT utilization might have been driven by the concern of underestimating insidious vascular complications in COVID-19 patients, especially during the year 2020, when little was known about the CNS complications induced by COVID-19 and the reliability and usability of COVID-19 tests were not so high. 

It is also only possible to consider the healthcare working environment in the COVID-19 era, which was burdened by a tense work atmosphere, high workloads, and stressful working conditions, all of which led to healthcare workers not being able to give their complete input [14,15].

These factors, associated with only a partial knowledge of the virus but a deep awareness of its wide range of action with multi-organ involvement (including the brain), might have led to an overuse of some healthcare resources, including neuroimaging, even in patients without significant neurological symptoms. 

These aspects, which can be interpreted as excesses of prudence, are, however, not exempt from problems both in economic terms for the healthcare system, for radioprotection issues related to exposure to ionizing radiation, and also for the overuse of contrast media (even if our study shows a limited use of contrast media in patients with renal insufficiencies), and the psychological pressure exerted on doctors can also increase the possibility of confirmation errors in the interpretation of false-positive CT scans. Our work had several limitations. The major limitation was its monocentric nature. Therefore, it was not possible to compare our results with those from other sites in Italy and other countries. Other limitations include the difference, in terms of length, of the three periods considered, with the vaccine period being shorter than the COVID-19 period and the control group being the longest period. Finally, our work is based solely on ED discharge diagnoses, and we could not record any discrepancies with our direct re-evaluation of the images.

## 5. Conclusions

CT is an invaluable diagnostic tool, and its benefits, if adequately indicated, far outweigh the risks associated with radiation, considering that modern equipment can capture excellent images with low doses of radiation. In any case, the 20% increase in skull CT examinations is notable if we also consider that COVID-19 patients are generally subjected to many other studies that involve the use of radiation (for example, X-rays and chest CT scans). [18].

A few years after the outbreak of the COVID-19 pandemic, the effects of the infection are still not fully known. Still, it is possible to hypothesize that the stress-inducing conditions to which all healthcare workers have been subjected are associated with the lack of knowledge regarding the infection, prompting them to use CT/contrast CT of the head more than usual.

## Figures and Tables

**Table 1:** Clinical characteristics of enrolled patients according to the date of emergency department access. The vaccine period (1 February 2021 to 30 September 2021) and COVID-19 period (1 March 2020 to 31 January 2021) are compared to the control period, which was defined to span a range of about two years (1 January 2018 to 29 February 2020). Values are presented as median [interquartile range] or number (percentage).

	All Cases *n* 167,549	Vaccine Period *n* 30,284	COVID-19 Period *n* 37,303	Control Period *n* 99,962	*p* Value
Age (years Q1–Q3)	60 (44–75)	60 (44–75)	61 (45–76)	59 (43–75)	<0.001
Sex (male %)	83,657 (49.9)	15,436 (51.0)	19,584 (52.5)	48,637 (48.7)	<0.001
ED Presentation					
High-priority triage (%)	11,311 (6.8)	2344 (7.7)	3150 (8.4)	5817 (5.8)	<0.001
Access by EMS (%)	32,715 (19.5)	6767 (22.3)	10,708 (28.7)	15,240 (15.2)	<0.001
Main symptoms					
Neurological deficit (%)	10,009 (6.0)	1899 (6.3)	2170 (5.8)	5940 (5.9)	0.038
Alt. consciousness (%)	3896 (2.3)	1006 (3.3)	1229 (3.3)	1661 (1.7)	<0.001
Epilepsy (%)	5082 (3.0)	623 (2.1)	756 (2.0)	3703 (3.7)	<0.001
Confusion (%)	4414 (2.6)	759 (2.5)	952 (2.6)	2703 (2.7)	0.090
Headache (%)	8834 (5.3)	1616 (5.3)	1615 (4.3)	5603 (5.6)	<0.001
Sensitivity disorders (%)	4024 (2.4)	852 (2.8)	732 (2.0)	2440 (2.4)	<0.001
Vertigo/dizziness (%)	5206 (3.1)	819 (2.7)	857 (2.3)	3530 (3.5)	<0.001
Malaise/fatigue (%)	18,557 (11.1)	3128 (10.3)	3681 (9.9)	11,748 (11.8)	<0.001
Vomit (%)	15,096 (9.0)	2641 (8.7)	2883 (7.7)	9572 (9.6)	<0.001
Syncope/pre-syncope (%)	9059 (5.4)	1436 (4.7)	1706 (4.6)	5917 (5.9)	<0.001
Clinical history—comorbidities					
Cerebrovascular disease (%)	4024 (2.4)	852 (2.8)	732 (2.0)	2440 (2.4)	<0.001
Peripheral vascular disease (%)	21,406 (12.8)	3679 (12.1)	4777 (12.8)	12,950 (13.0)	0.001
Hypertension (%)	40,592 (24.2)	7510 (24.8)	9404 (25.2)	23,678 (23.7)	<0.001
Lipid disorders (%)	13,317 (7.9)	2571 (8.5)	3032 (8.1)	7714 (7.7)	<0.001
History of CAD (%)	19,707 (11.8)	3272 (10.8)	4346 (11.7)	12,089 (12.1)	<0.001
Diabetes (%)	15,399 (9.2)	2721 (9.0)	3566 (9.6)	9112 (9.1)	0.016
Chronic kidney disease (%)	12,228 (7.3)	2208 (7.3)	2736 (7.3)	7284 (7.3)	0.954
Anticoagulant therapy (%)	5258 (3.1)	866 (2.9)	1126 (3.0)	3266 (3.3)	0.001
Antiplatelet therapy (%)	12,292 (7.3)	1951 (6.4)	2432 (6.5)	7909 (7.9)	<0.001
Outcomes					
Head CT scan in ED (%)	24,566 (14.7)	4414 (14.6)	6058 (16.2)	14,094 (14.1)	<0.001
Contrast Head CT in ED (%)	2915 (1.7)	678 (2.2)	704 (1.9)	1533 (1.5)	<0.001
Cerebral venous thrombosis (%)	36 (2.14/10^4^) *	13 (4.29/10^4^) *	7 (1.8/10^4^) *	16 (1.6/10^4^) *	0.018
Acute Stroke (%)	3415 (2.0)	399 (1.3)	471 (1.3)	2545 (2.5)	<0.001
Cerebral hemorrhage (any) (%)	1599 (1.0)	249 (0.8)	358 (1.0)	992 (1.0)	0.028
COVID-19-positive (%)	4668 (2.8)	2306 (7.6)	2301 (6.2)	/	<0.001

* Values are reported as cases per 10^4^ patients. Abbreviations: EMS—Emergency Medical Services; CAD—coronary artery disease.

**Table 2:** Study variables in patients that were prescribed a head CT scan in the ED either with or without a contrast agent.

	Head CT Scan in ED *n* 25,436	Controls *n* 142,091	Unadjusted *p* Value	Odds Ratio [95% CI] for Head CT Prescription in ED	Adjusted *p* Value
Age (years)	68 (50–81)	58 (43–74)	<0.001	1.011 [1.010–1.012]	<0.001
Sex (male %)	12,553 (49.3)	71,104 (50.0)	0.043	1.064 [1.027–1.102]	0.001
Emergency department presentation					
Presentation period:					
Control years (%)	14,676 (57.7)	85,286 (60.0)		Reference	
COVID-19 period (%)	6187 (24.3)	31,116 (21.9)		1.182 [1.132–1.234]	<0.001
Vaccine period (%)	4576 (18.0)	25,708 (18.1)	<0.001	0.975 [0.930–1.023]	0.302
High-priority triage (%)	5248 (20.6)	6063 (4.3)	<0.001	3.867 [3.661–4.085]	<0.001
Access by EMS (%)	11,021 (43.3)	21,694 (15.3)	<0.001	2.685 [2.579–2.796]	<0.001
Main symptoms					
Neurological deficit (%)	6747 (26.5)	3262 (2.3)	<0.001	12.907 [12.215–13.639]	<0.001
Alteration in consciousness (%)	2729 (10.7)	1167 (0.7)	<0.001	8.665 [7.960–9.432]	<0.001
Epilepsy (%)	2132 (8.4)	2950 (2.1)	<0.001	4.012 [3.713–4.336]	<0.001
Confusion (%)	3344 (13.1)	1070 (0.8)	<0.001	17.267 [15.875–18.782]	<0.001
Headache (%)	5132 (20.2)	3702 (2.6)	<0.001	14.389 [13.591–15.322]	<0.001
Sensitivity disorders (%)	1595 (6.3)	2429 (1.7)	<0.001	3.090 [2.831–3.373]	<0.001
Vertigo/dizziness (%)	2968 (11.7)	2238 (1.6)	<0.001	8.349 [7.769–8.973]	<0.001
Malaise/fatigue (%)	4468 (17.6)	14,089 (9.9)	<0.001	1.943 [1.852–2.039]	<0.001
Vomit (%)	2727 (10.7)	12,369 (8.7)	<0.001	1.126 [1.061–1.196]	<0.001
Syncope/pre-syncope (%)	3975 (15.6)	5084 (3.6)	<0.001	4.228 [3.992–4.479]	<0.001
Clinical history—comorbidities					
Cerebrovascular disease (%)	2777 (10.9)	6928 (4.9)	<0.001	1.433 [1.337–1.535]	<0.001
Peripheral vascular disease (%)	3107 (12.2)	18,299 (12.9)	0.004	0.547 [0.516–0.580]	<0.001
Hypertension (%)	9188 (36.1)	31,404 (22.1)	<0.001	1.314 [1.260–1.370]	<0.001
Lipid disorders (%)	2507 (9.9)	10,810 (7.6)	<0.001	0.981 [0.921–1.044]	0.540
History of CAD (%)	2978 (11.7)	16,729 (11.8)	0.765		
Diabetes (%)	3006 (11.8)	12,393 (8.7)	<0.001	0.987 [0.931–1.047]	0.674
Chronic kidney disease (%)	1217 (4.8)	11,011 (7.7)	<0.001	0.594 [0.550–0.641]	<0.001
Anticoagulant therapy (%)	1246 (4.9)	4012 (2.8)	<0.001	1.454 [1.330–1.591]	<0.001
Antiplatelet therapy (%)	2807 (11.0)	9485 (6.7)	<0.001	1.266 [1.189–1.348]	<0.001

Abbreviations: EMS—emergency medical services; CAD—coronary artery disease.

**Table 3:** Study variables in patients that were prescribed a head CT angiography scan in the emergency department. Controls were considered patients that did not receive a head CT scan or received a non-contrast CT scan.

	Head CT Angiography *n* 2923	Controls *n* 164,604	Unadjusted *p* Value	Odds Ratio [95% CI] for Head CT Prescription in ED	Adjusted *p* Value
Age (years)	59 (43–75)	71 (55–81)	<0.001	1.007 [1.004–1.009]	<0.001
Sex (male %)	1455 (49.8)	82,202 (49.9)	0.853		
ED presentation					
Presentation period:					
Control years (%)	1537 (52.6)	98,425 (59.8)		Reference	
COVID-19 period (%)	707 (24.2)	36,596 (22.2)		1.107 [0.997–1.229]	0.057
Vaccine period (%)	680 (23.3)	29,604 (18.0)	<0.001	1.339 [1.197–1.498]	<0.001
High-priority triage (%)	1745 (59.7)	9566 (5.8)	<0.001	15.542 [14.035–17.212]	<0.001
Access by EMS (%)	1763 (60.3)	30,952 (18.8)	<0.001	1.674 [1.515–1.849]	<0.001
Main symptoms					
Neurological deficit (%)	1672 (57.2)	8337 (5.1)	<0.001	11.267 [10.311–12.312]	<0.001
Alteration in consciousness (%)	310 (10.6)	3586 (2.2)	<0.001	1.268 [1.091–1.473]	0.002
Epilepsy (%)	172 (5.9)	4910 (3.0)	<0.001	0.781 [0.646–0.945]	0.011
Confusion (%)	309 (10.6)	4105 (2.5)	<0.001	1.659 [1.424–1.934]	<0.001
Headache (%)	509 (17.4)	8325 (5.1)	<0.001	5.212 [4.591–5.918]	<0.001
Sensitivity disorders (%)	203 (6.9)	3821 (2.3)	<0.001	2.044 [1.715–2.436]	<0.001
Vertigo/dizziness (%)	181 (6.2)	5025 (3.1)	<0.001	2.098 [1.750–2.436]	<0.001
Malaise/fatigue (%)	361 (12.3)	18,196 (11.1)	0.027	1.473 [1.296–1.674]	<0.001
Vomit (%)	256 (8.8)	14,840 (9.0)	0.627		
Syncope/pre-syncope (%)	225 (7.7)	8834 (5.4)	<0.001	0.863 [0.734–1.014]	0.074
Clinical history—comorbidities					
Cerebrovascular disease (%)	387 (13.2)	9318 (5.7)	<0.001	1.209 [1.052–1.390]	0.008
Peripheral vascular disease (%)	317 (10.8)	21,089 (12.9)	0.002	0.376 [0.179–0.787]	0.009
Hypertension (%)	1121 (38.3)	39,471 (24.0)	<0.001	1.282 [1.164–1.412]	<0.001
Lipid disorders (%)	226 (7.7)	13,091 (8.0)	0.659		
History of CAD (%)	309 (10.6)	19,398 (11.8)	0.043	1.681 [0.795–3.554]	0.174
Diabetes (%)	336 (11.5)	15,063 (9.1)	<0.001	0.869 [0.756–1.000]	0.049
Chronic kidney disease (%)	68 (2.3)	12,160 (7.4)	<0.001	0.355 [0.274–0.460]	<0.001
Anticoagulant therapy (%)	216 (7.4)	5042 (3.1)	<0.001	1.712 [1.432–2.048]	<0.001
Antiplatelet therapy (%)	328 (11.2)	11,964 (7.3)	<0.001	1.095 [0.949–1.264]	0.214

Abbreviations: EMS—emergency medical services; CAD—coronary artery disease.

## Data Availability

All data generated or analyzed during this study are included in this article. Further enquiries can be directed to the corresponding author.

## Supplementary Materials

The following supporting information can be downloaded at: https://www.mdpi.com/article/10.3390/life14020264/s1, Table S1: The table presents absolute and relative frequencies of the three main diagnoses in relation to symptoms, in the three period: control, COVID, and vaccine period; Table S2: The table shows extended analysis from Logistic Regression Results For Head CT Scan Prescription showed in Table 2 in the main manuscript; Table S3: The table shows extended analysis from Logistic Regression Results For Contrast Head CT Scan Prescription showed in Table 3 in the main manuscript.
