# Supplementary material for: Head CT Scans in the Emergency Department during the COVID-19 Pandemic: Use or Overuse?
Source: Life (Basel). 2024 Feb 17;14(2):264. doi: 10.3390/life14020264 (PMC10890022; doi:10.3390/life14020264)
Supplement: Supplementary file 1 [file life-14-00264-s001.zip › life-2796295-supplementary.pdf]

Table S1. The table presents absolute and relative frequencies of the three main diagnoses in relation to symptoms, in the three period: control, covid, and vaccine period.

Control Period (2018-2019)			
**Symptom**	**Cerebral vein thrombosis**	**Ischemic Stroke**	**Cerebral Hemorrhage**
Neurological deficit	6/16 (37.5%)	1052/1576 (66.8%)	237/985 (24.1%)
Alt. Consciousness	0/16 (0)	91/1576 (5.8%)	129/985 (13.1%)

COVID PERIOD			
**Symptom**	**Cerebral vein thrombosis**	**Ischemic Stroke**	**Cerebral Hemorrhage**
Neurological deficit	4/7 (57.1%)	287/470 (61.1%)	88/358 (24.6%)
Alt. Consciousness	1/7 (14.3%)	52/470 (11.1%)	87/358 (24.3%)

VACCINE PERIOD			
**Symptom**	**Cerebral vein thrombosis**	**Ischemic Stroke**	**Cerebral Hemorrhage**
Neurological deficit	2/13 (15.4%)	226/398 (56.8%)	72/249 (28.9%)
Alt. Consciousness	1/13 (7.7%)	60/398 (15.1%)	46/249 (18.5%)

Table S2. The table shows extended analysis from Logistic Regression Results For Head CT Scan Prescription showed in Table 2 in the main manuscript.

Logistic Regression Results For Head CT Scan Prescription (Table 2 in the main manuscript)

**Variable**	**Beta**	**Standard Error**	**Wald Statistic**	***p*-value**	**Odds Ratio**	**95% CI (low)**	**95% CI (High)**
Age	.011	.001	399.718	<0.001	1.011	1.010	1.012
Sex (M)	.067	.018	13.835	<0.001	1.064	1.032	1.108
Control period			69.232	<0.001	*Reference*		
• COVID-19 period	.167	.022	58.382	<0.001	1.182	1.132	1.234
• Vaccine Period	-.025	.024	1.066	0.302	.975	.930	1.023
High priority Triage	1.353	.028	2350.993	<0.001	3.867	3.661	4.085
Access by EMS	.988	.021	2298.139	<0.001	2.685	2.579	2.796
Neurological deficit	2.558	.028	8276.366	<0.001	12.907	12.215	13.639
Alteration of Consciousness	2.159	.043	2488.131	<0.001	8.665	7.960	9.432
Epilepsy	1.389	.040	1230.057	<0.001	4.012	3.713	4.336
Confusion	2.849	.043	4410.431	<0.001	17.267	15.875	18.782
Headache	2.666	.029	8402.121	<0.001	14.389	13.591	15.233
Sensitivity disorders	1.128	.045	637.347	<0.001	3.090	2.831	3.373
Vertigo/dizziness	2.122	.037	3333.179	<0.001	8.349	7.769	8.973
Malaise/fatigue	.664	.025	727.881	<0.001	1.943	1.852	2.039
Vomit	.119	.031	15.049	<0.001	1.126	1.061	1.196
Syncope/pre-syncope	1.442	.029	2413.547	<0.001	4.228	3.992	4.479
Cerebrovascular disease	.360	.035	104.273	<0.001	1.433	1.337	1.535
Peripheral vascular disease	-.603	.030	409.842	<0.001	.547	.516	.580
Hypertension	.273	.021	164.038	<0.001	1.314	1.260	1.370
Lipid disorders	-.020	.032	.376	0.540	.981	.921	1.044
Diabetes (no)	.013	.030	.177	0.674	1.013	.955	1.074
Chronic kidney disease	-.521	.039	175.696	<0.001	.594	.550	.641
Anticoagulant therapy	.375	.046	66.832	<0.001	1.454	1.330	1.591
Antiplatelet therapy	.236	.032	53.737	<0.001	1.266	1.189	1.348
Constant	-4.083	.048	7109.631	<0.001	.017		

Table S3. The table shows extended analysis from Logistic Regression Results For Contrast Head CT Scan Prescription showed in Table 3 in the main manuscript

Logistic regression results. Contrast Head CT scan prescription (Main mauscript Table 3)

**Variable**	**Beta**	**Standard Error**	**Wald Statistic**	***p*-value**	**Odds Ratio**	**95% CI (low)**	**95% CI (High)**
Age	.007	.001	25.838	.000	1.007	1.004	1.009
Control period			30.709	.000			
• COVID-19 period	.101	.053	3.607	.057	1.107	.997	1.229
• Vaccine Period	.301	.054	30.691	.000	1.339	1.197	1.498
High priority Triage	2.740	.052	2780.510	.000	15.542	14.035	17.212
Access by EMS	.518	.051	104.141	.000	1.674	1.515	1.849
Neurological deficit	2.421	.045	2865.670	.000	11.252	10.297	12.294
Alteration of Consciousness	.240	.076	9.848	.002	1.271	1.094	1.477
Epilepsy	-.248	.097	6.540	.011	.780	.645	.944
Confusion	.507	.078	42.109	.000	1.660	1.425	1.935
Headache	1.662	.064	671.063	.000	5.270	4.648	5.977
Sensitivity disorders	.712	.089	63.417	.000	2.038	1.710	2.428
Vertigo/dizziness	.750	.092	66.335	.000	2.117	1.768	2.536
Malaise/fatigue	.388	.065	35.405	.000	1.475	1.297	1.676
Syncope/pre-syncope	-.143	.082	2.990	.084	.863	.734	1.014
Cerebrovascular disease	.190	.071	7.169	.007	1.210	1.052	1.391
Peripheral vascular disease	-.981	.377	6.765	.009	.375	.179	.785
Hypertension	.251	.049	26.014	.000	1.285	1.167	1.415
History of CAD	.519	.382	1.846	.174	1.680	.795	3.551
Diabetes	-.141	.071	3.909	.048	.868	.755	.999
Chronic kidney disease	-1.033	.132	61.069	.000	.356	.275	.461
Anticoagulant therapy	.538	.091	34.695	.000	1.712	1.432	2.048
Antiplatelet therapy	.091	.073	1.546	.214	1.095	.949	1.264
Constant	-6.385	.091	4915.226	.000	.002		
